# Optimizing recombinant protein expression via automated induction profiling in microtiter plates at different temperatures

**DOI:** 10.1186/s12934-017-0832-4

**Published:** 2017-11-28

**Authors:** Martina Mühlmann, Eva Forsten, Saskia Noack, Jochen Büchs

**Affiliations:** AVT-Chair for Biochemical Engineering, RWTH Aachen University, Forckenbeckstraße 51, 52074 Aachen, Germany

**Keywords:** Induction profiling, Recombinant protein, Temperature, *E. coli*, High-throughput, Microtiter plate, BioLector

## Abstract

**Background:**

*Escherichia coli* (*E. coli*) is the most abundant expression host for recombinant proteins. The production efficiency is dependent on a multitude of parameters. Therefore, high-throughput applications have become an increasingly frequent technique to investigate the main factors. Within this study, the effects of temperature, induction time and inducer concentration on the metabolic state and the product formation were extensively examined. Induction profiling of *E. coli* Tuner(DE3) pRhotHi-2-EcFbFP was performed in 48-well Flowerplates and standard 96-well plates using a robotic platform. In parallel shake flask cultivations, the respiration activity of the microorganisms was analyzed. Therefore, two online-monitoring systems were applied: the BioLector for microtiter plates and the RAMOS-device for shake flasks. The impact of different induction conditions on biomass and product formation as well as on the oxygen transfer rate was surveyed.

**Results:**

Different optimal induction conditions were obtained for temperatures of 28, 30, 34, and 37 °C. The best inducer concentrations were determined to be between 0.05 and 0.1 mM IPTG for all investigated temperatures. This is 10–20 times lower than conventional guidelines suggest. The induction time was less relevant when the correct inducer concentration was chosen. Furthermore, there was a stronger impact on growth and respiration activity at higher temperatures. This indicated a higher metabolic burden. Therefore, lower IPTG concentrations were advantageous at elevated temperatures. Very similar results were obtained in standard 96-well plates.

**Conclusion:**

Two online-monitoring systems were successfully used to investigate the optimal induction conditions for the *E. coli* Tuner(DE3) pRhotHi-2-EcFbFP strain (*lacY* deletion mutant) at four different temperatures. The experimental effort was reduced to a minimum by integrating a liquid handling robot. To reach the maximum product formation, a detailed induction analysis was necessary. Whenever the cultivation temperature was changed, the induction conditions have to be adapted. Due to the experimental options provided by the BioLector technology, it was found that the higher the cultivation temperature, the lower the inducer concentration that has to be applied.

**Electronic supplementary material:**

The online version of this article (10.1186/s12934-017-0832-4) contains supplementary material, which is available to authorized users.

## Background

In 1982, the recombinant protein technology achieved the great breakthrough with Humulin, a recombinant human insulin expressed in *Escherichia coli* (*E. coli*), which was the first approved biopharmaceutical [1]. Since that time, more than 400 recombinant proteins were introduced to the market [2]. *E. coli* is still the most popular host organism for heterologous protein production due to its well-known genetics, high growth rates on inexpensive media, and a wide range of possible expression vectors [3].

Recombinant protein production always implicates a perturbation of the host cell metabolism, which typically leads to lower biomass growth rates [4–6]. This stems from high maintenance requirements for the replication of inserted plasmids and from the resources needed for the transcription of target genes [7–9]. Particularly, plasmids with high copy numbers can overburden the cell and induce a metabolic collapse, which results in low product yields or even cell death [10–12]. Thus, a good balance of biomass growth and product formation associated with unavoidable cell stress has to be found to achieve high productivity. Therefore, many parameters have to be individually optimized. This involves not only the choice of vector and promoter system, but also composition of the medium, oxygen supply, cultivation temperature and induction conditions [3, 10, 13–17].

The reduction of cultivation temperature is one of the common approaches to prevent formation of inclusion bodies by correct folding of the molecule [18–20]. Unfortunately, this comes along with prolonged cultivation times. A fast approach to investigate temperature effects in microtiter plates is through temperature profiling as described by Kunze et al. [21].

One of the most frequently used expression systems is the *E. coli* T7 system under control of the *lac* operon, present in pET and pRhot vectors. The expression of the target protein can be induced with lactose or the non-degradable analog isopropyl β-d-thiogalactopyranoside (IPTG) [22, 23]. Such inducible expression systems allow the precise and adjustable separation of growth and production phases by the variation of inducer concentration and induction time. However, the large space available for optimization is also coupled to high experimental effort. This can be handled with miniaturized cultivations in high-throughput applications. The fast detection of optimized induction conditions can be conducted on robotic platforms [24].

Miniaturization of culture scale may impede access to process parameters and sufficient oxygen supply. Oxygen limited cultivations should be avoided to achieve reproducible results and enable easy scale-up. Moreover, insufficient oxygen supply mostly leads to altered metabolism and lower product formation [25–27]. A good strategy is to use special 48-well flower-shaped microtiter plates incubated in a BioLector device to detect biomass and fluorescent molecules via optical measurements. The flower shape of the wells serve as baffles allowing oxygen transfer rates (OTR) of up to 100 mmol/L/h [28, 29].

Several studies have shown the results of induction profiles in 48-well Flowerplates but either at single temperatures [24, 30–32] or with few data points [33, 34]. The aim of this study was to comprehensively investigate the effect of temperature on optimal induction conditions. Therefore, the fluorescent model protein FbFP was expressed in *E. coli* Tuner(DE3) under control of the T7 promoter. This strain has a *lacY* deletion to prevent the active transport of IPTG into the cell. Instead, IPTG enters the cell solely via concentration-dependent diffusion [35]. Complete induction profiles with variations of IPTG concentrations and times of induction were performed in 48-well Flowerplates. The robotic platform RoboLector consisting of a liquid handling system and a BioLector device was used to enable an automated induction. During cultivation, biomass concentration and product formation were detected via scattered light and fluorescence measurement. Oxygen transfer rates were observed in simultaneous shake flask cultivations using the Respiration Activity Monitoring System (RAMOS)-device and compared to the results obtained in microtiter plates.

## Methods

### Microorganism

For all experiments, the strain *E. coli* Tuner(DE3) pRhotHi-2-EcFbFP was used. The Tuner(DE3) strains are *lacY* deletion mutants of BL21; therefore, no lactose permease is produced. The entry of IPTG is solely regulated by concentration-dependent diffusion which leads to adjustable levels of homogenous protein expression throughout the entire population [35]. A copy of the T7 polymerase gene controlled by the *lac*UV5 promoter is chromosomally integrated. The plasmid pRhotHi-2 is provided with a T7 promoter to allow inducible high-level expression of heterologous proteins [23]. The recombinant protein EcFbFP is an (FMN)-binding fluorescent protein, which is codon-optimized for the expression in *E. coli* and whose maturation is, compared to GFP, independent of oxygen supply [36].

### Shake flask cultivations in RAMOS device

The in-house manufactured RAMOS is a device used for online-monitoring of the OTR, the carbon dioxide transfer rate (CTR) and the respiratory quotient (RQ) in up to eight shake flasks. The OTR is a suitable parameter to quantify the physiological state of a culture of aerobic microorganisms [37]. Commercial versions of the RAMOS device can be purchased from Kühner AG, Birsfelden, Switzerland or HiTec Zang GmbH, Herzogenrath, Germany. All shake flask cultivations were conducted in 250 mL shake flasks and cultivated on an orbital shaker (ISF1-X, Kühner AG, Birsfelden, Switzerland) at a shaking frequency of 350 rpm and a shaking diameter of 50 mm.

### Microtiter plate cultivations in the BioLector device

The BioLector is an online-monitoring system for the measurement of biomass and product formation in microtiter plates [28, 38]. An optical fiber is moved sequentially below each well of a shaken microtiter plate while light is transmitted from a light source into the sample. Depending on the applied optical filter or monochromator position, the backscattered light (indicator for biomass) or the fluorescence of the sample (indicator for fluorescent products) can be detected. In this work, a commercially available BioLector (m2p-Labs, Baesweiler, Germany) was used for the measurement of scattered light, FbFP-fluorescence and DOT. For DOT measurements, Flowerplates with optodes were used (MTP-48-BO, m2p-Labs, Baesweiler, Germany). The corresponding excitation and emission wavelength as well as the gain factors are specified in Table 1. 48-well and 96-well microtiter plate cultivations were performed at a shaking frequency of 1400 and 1000 rpm, respectively. The shaking diameter was always 3 mm.

**Table 1:** Applied excitation and emission wavelength for BioLector cultivations

Optical signal	Excitation (nm)	Emission (nm)	Gain (−)
Scattered light	620	–	20
FbFP	450	492	60
DOT	520	600	83

The BioLector is part of the so-called RoboLector platform which combines the BioLector technology with a liquid handling system. It was already introduced by Huber et al. [24] and extended by Mühlmann et al. [39]. The robot enables the automation of many process steps and is used in this work for the automated induction of cultures.

### Growth media

Two different growth media were used for cultivation. The first preculture was incubated in complex terrific broth (TB) [40]. The medium was composed of 12 g/L tryptone (Lot Number: 134209944), 24 g/L yeast extract (Lot Number: 314216947), 12.54 g/L K_2_HPO_4_, 2.31 g/L KH_2_PO_4_ and 5 g/L glycerol dissolved in deionized water. All ingredients were purchased from Roth, Karlsruhe, Germany. The pH-value was 7.2 ± 0.2 without adjustment. The second preculture and the main culture were carried out in modified Wilms-MOPS mineral medium according to Wilms et al. [41]. The mineral medium consisted of 20 g/L glucose, 6.98 g/L (NH_4_)_2_SO_4_, 3 g/L K_2_HPO_4_, 2 g/L Na_2_SO_4_, 0.2 M (*N*-Morpholino)-propanesulfonic acid (MOPS), 0.5 g/L MgSO_4_·7H_2_O, 0.01 g/L thiamine hydrochloride, 1 mL/L trace element solution [0.54 g/L ZnSO_4_·7H_2_O, 0.48 g/L CuSO_4_·5H_2_O, 0.3 g/L MnSO_4_·H_2_O, 0.54 g/L CoCl_2_·6H_2_O, 41.76 g/L FeCl_3_·6H_2_O, 1.98 g/L CaCl_2_·2H_2_O, 33.4 g/L Na_2_EDTA·2H_2_O (Titriplex III)]. The pH-value was adjusted to 7.5 using 1 M NaOH. Both media were supplemented to obtain a final concentration of 50 µg/mL kanamycin from a 1000-fold concentrated stock solution prior to cultivation.

For induction, IPTG was dissolved in a pure basic Wilms-MOPS mineral medium (without glucose, trace elements, magnesium sulfate, thiamine and kanamycin). Different concentrated stock solutions were prepared to enable the final correct IPTG concentrations (0–1 mM) in the respective microtiter plate well.

### Precultures

All experiments started with two precultivation steps in shake flasks. For the first preculture, 10 mL of TB-medium were inoculated to an initial optical density of 0.05 (OD_600_) from cryogenically preserved cultures and incubated over night at 30 °C. For the second preculture, 8 mL of the modified Wilms-MOPS mineral medium were inoculated to an initial optical density of 0.2 (OD_600_) from the first preculture and incubated at 37 °C in a RAMOS device until the OTR reached a value between 20 and 40 mmol/L/h.

### Main cultivation

The main cultivations were carried out simultaneously in RAMOS and BioLector devices at temperatures of 28, 30, 34, and 37 °C. To enable equal starting conditions in shake flasks and microtiter plates, a *mastermix* consisting of Wilms-MOPS mineral medium inoculated with cells from the second preculture was prepared. The initial optical density (OD_600_) was set to 0.1. The *mastermix* was thereupon distributed among the cultivation systems. The shake flask cultivation conditions were identical to the second preculture. Microtiter plate cultivations were conducted in 48-well Flowerplates (m2p-Labs, Baesweiler, Germany) and 96-well plates (black-clear, BD-Bioscience, USA). Each well was filled either with 800 µL (48-well) or 200 µL (96-well) *mastermix* and sealed with a gas permeable perforated silicone layer (F-GPRS48-10, m2p-Labs, Baesweiler, Germany) or a gas permeable membrane (AeraSeal, Excel-Scientific, CA, USA).

### Induction profiling

During the main cultivation, the induction was carried out automatically for microtiter plate cultivations by the liquid handling system (Hamilton STAR, Hamilton Robotics, Martinsried, Germany) as part of the RoboLector platform and manually for shake flask cultivations. An appropriate concentrated IPTG-gradient was provided in a single 96-well microtiter plate (flat-bottom, Roth, Karlsruhe, Germany) sealed with X-pierce film (XP-25, Excel Scientific, CA, USA). This plate was located outside of the BioLector on the RoboLector deck at room temperature. Within the programmed RoboLector method, the user can individually select the columns to be induced, the induction volume and time. For induction profiling, 20 µL (48-well) or 10 µL (96-well) of the provided IPTG solution were transferred column by column at predefined times by the liquid handling system into the microtiter plate standing inside the BioLector device. By the end, the IPTG concentration in each well was between 0–1 mM. For induction with 0 mM IPTG, pure basic Wilms-MOPS mineral medium (without glucose, trace elements, magnesium sulfate, thiamine and kanamycin) instead of IPTG, was used.

### Offline analysis

Standard optical density (OD_600_) was determined manually for the calibration curve (Additional file 1: Figure S5) via a Genesys 20 photometer (Thermo Scientific, Dreieich, Germany) in 1.5 mL microcuvettes (PS, Plastibrand, Roth, Karlsruhe, Germany). For values higher than 0.4 the samples were appropriately diluted with 0.9% [m/v] NaCl solution. Osmolality was measured by a cryoscopic osmometer (Osmomat 030, Gonotec GmbH, Berlin, Germany).

### Calibration curve

To allow the conversion of scattered light, measured by the RoboLector device, into standard OD_600_, calibration curves for 48-well and 96-well microtiter plates were generated. A non-induced main culture from the stationary phase was utilized. The cell culture was centrifuged at 18,000*g* for 10 min, the supernatant was removed and the cell pellet was resuspended in Wilms-MOPS mineral medium (without glucose) to adjust OD_600_ values from 0.1 to 12. Cavities of a 48-well Flowerplate (singular determination of samples) and 96-well microtiter plate (triplicate determination of samples) were filled with 800 or 200 µL, respectively. Scattered light was measured at temperatures of 28, 30, 34, and 37 °C, applying the BioLector system with the parameters listed in Table 1. Data were determined from at least five consecutive measurement cycles. The shaking frequencies used were identical to the cultivation conditions. The data were fitted using linear regression with the software Origin 9.0. The results are presented in Additional file 1: Figure S5. These fits were applied to calculate the OD_600_ at the respective time of induction for all induction profiles (upper x-axis of Figs. 2, 4, 5 and Additional file 1: Figures S1–S2). The scattered light mean of all cultivations that have not been induced up to this time were used to obtain the respective OD_600_.

## Results and discussion

### Comparable oxygen supply in microtiter plates and shake flasks

**Fig. 1:**
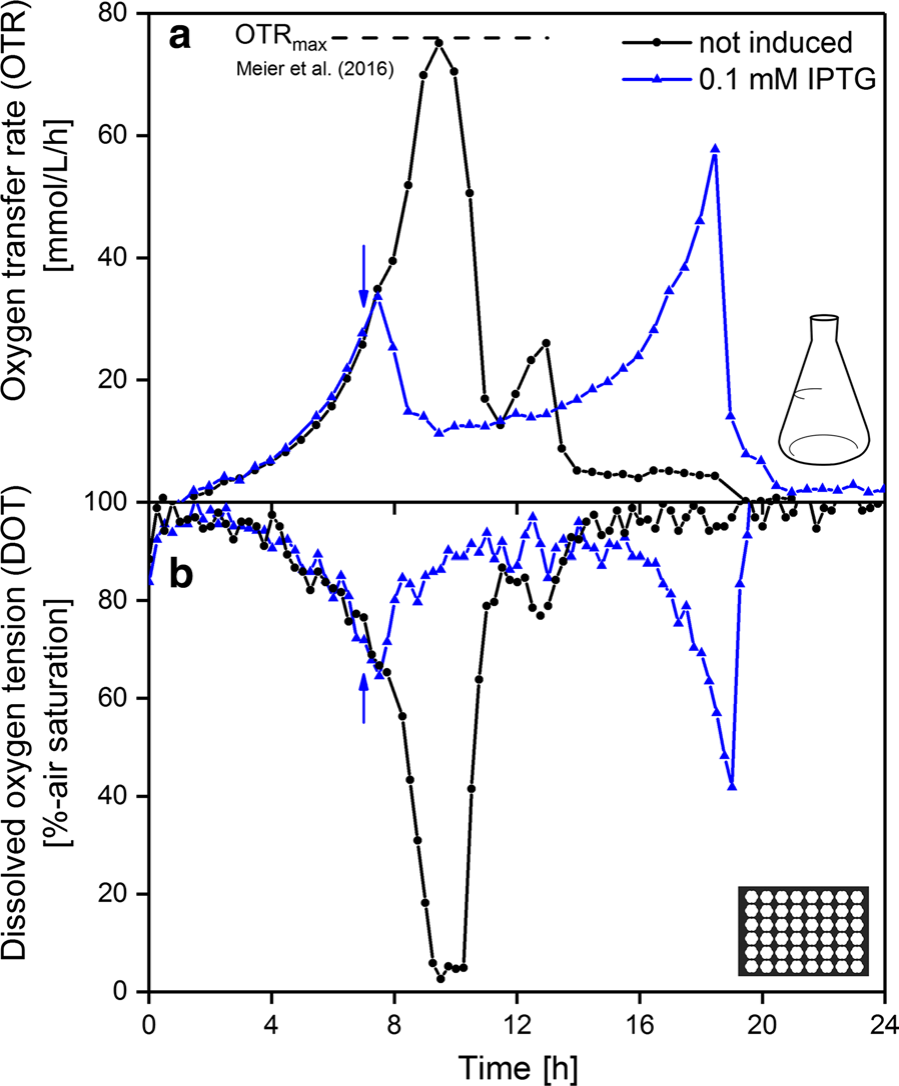
Comparison of oxygen transfer rate (OTR, measured in shake flasks) and dissolved oxygen tension (DOT, measured in microtiter plates) measured by a RAMOS and a BioLector device, respectively. A *mastermix* of medium and cells was used to start the experiment in both systems simultaneously. The non-induced cultures served as references. The induction for the induced cultures took place after 7 h (blue arrows) by the addition of IPTG (0.1 mM final concentration in cultures). The maximum oxygen transfer capacity (OTR_max_) was calculated after Meier et al. [46] (dashed line). Cultivation conditions for *E. coli* Tuner(DE3)/pRhotHi-2-EcFbFP in RAMOS device (**a**): 8 mL Wilms-MOPS mineral medium in each 250 mL flask, shaking frequency 350 rpm, shaking diameter 50 mm. Cultivation in the BioLector device (**b**): 800 µL Wilms-MOPS mineral medium per well in a 48-Flowerplate with DOT optodes, sealed with a sandwich membrane (m2p-labs), 37 °C, shaking frequency 1400 rpm and shaking diameter 3 mm

The combination of RAMOS and BioLector online-monitoring technologies provide broad information of investigated cultivations. However, two different reactor types, shake flasks and microtiter plates, have to be taken into account. To allow the simultaneous evaluation of both measurements, comparable cultivation conditions are required. One approach is to ensure identical oxygen supply in both fermentation systems [42, 43]. To avoid the production of anaerobic side products and to increase the recombinant protein yield, unlimited oxygen conditions are favored. The filling volume and shaking frequency can serve as parameters to adjust the maximum oxygen transfer capacity (OTR_max_) [44]. The upper limit of shaking frequency for shake flasks on a conventional shaker is typically 300–350 rpm at a shaking diameter of 50 mm. Furthermore, low filling volumes are associated with an increased ratio of evaporated water per applied culture volume. Therefore, the shaking frequency and the filling volume were set to 1400 rpm/350 rpm and 800 µL/8 mL for microtiter plates and shake flasks, respectively. To verify the comparability of both systems with these settings, *E. coli* Tuner(DE3) EcFbFP was cultured exemplarily under non-induced and induced cultivation conditions. The oxygen transfer rates were measured within a RAMOS device and DOTs were measured in a 48-well Flowerplate featuring optodes (MTP-48-BO, m2p-Labs, Baesweiler, Germany) using the BioLector device (m2p-Labs, Baesweiler, Germany). The OTR (measured in shake flasks) and DOT (measured in microtiter plates) during the simultaneous cultivation of *E. coli* Tuner(DE3) EcFbFP in RAMOS and BioLector devices are shown in Fig. 1a, b, respectively. Both parameters describe the oxygen supply of a culture and are, therefore, suitable for a comparison of both cultivation systems. The progress of OTR over time in the shake flask for the non-induced culture is characteristic for *E. coli* and well known from previous studies [42, 45]. The two peaks reflect increased respiration due to glucose consumption during the first 11 h of cultivation, followed by acetate consumption at around 11–14 h. The DOT in the microtiter plate runs entirely inverse to the OTR, leading to two local minima. The DOT describes the same phenomenon as the OTR, since decreasing dissolved oxygen availability in the surrounding media is the consequence of high oxygen consumption of the organism. This demonstrates the comparability of microtiter plate and shake flask cultivations under the chosen cultivation conditions. The measurement interval of DOT (15 min) was shorter than that for the OTR (30 min), leading to a higher data density. A short oxygen limitation of around 1 h can be detected, shortly before the OTR drops and the DOT rises. Although the DOT does not reach 0%, this is clearly an oxygen limitation. Low oxygen tensions cannot sufficiently be resolved with the used optical sensor spot (accuracy according to manufacturer ± 5%). The maximum reached OTR lies at 76 mmol/L/h. This agrees with the calculated OTR_max_ according to Meier et al. [46]. The osmolality, which is required to calculate the OTR_max_, was determined in an additional experiment (data not shown), where samples were taken every hour. The osmolality decreased from 0.69 Osmol/kg at the beginning of the cultivation to 0.52 Osmol/kg at the end of the cultivation. During the critical time at around 9–11 h, the osmolality reached a value of around 0.62 Osmol/kg, leading to the calculated OTR_max_ value. This is the first hint of a short oxygen limitation and it is confirmed when looking at the slope of the OTR curve. It is clearly visible that a higher OTR would have been reached when the increase in OTR would not be limited by the maximum oxygen transfer capacity of the shake flask under these cultivation conditions. Hence, a short oxygen limitation is present for both cultivation systems. This was accepted since induced cultures typically do not reach such high OTR values. This was proven through a culture which was induced after 7 h with 0.1 mM IPTG. The OTR and DOT differ largely from the non-induced culture. The oxygen consumption decreases more or less directly after induction and remains at a low plateau afterwards. At the end of the cultivation, the OTR increases again, but no oxygen limitation occurres throughout the cultivation. This can be confirmed by the progress of DOT as the curve never falls below 40% air saturation.

### Induction profiling covering conventional conditions

**Fig. 2:**
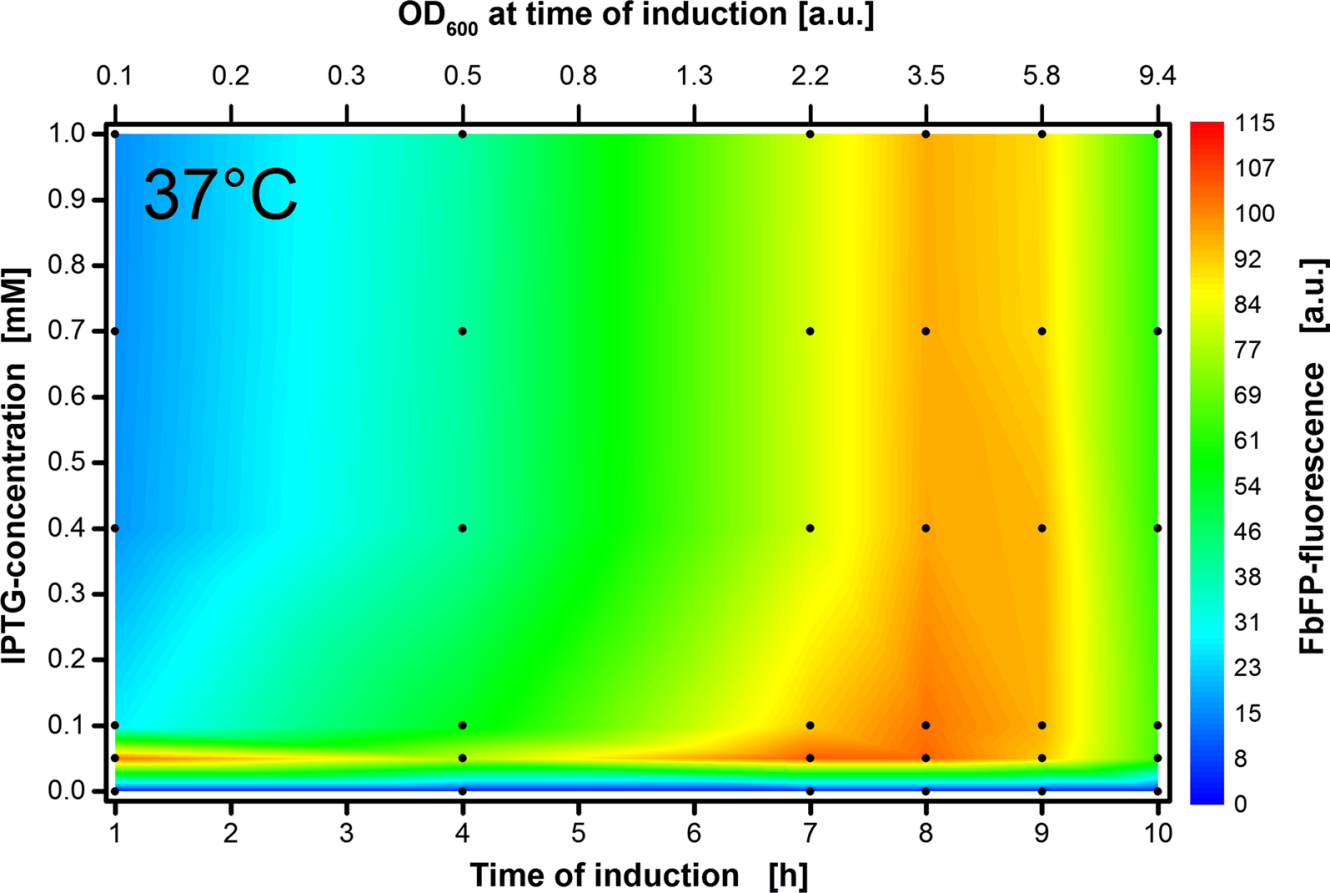
Induction profile with automated addition of 0–1 mM IPTG solution after 1–10 h of cultivation time. Colors from blue to red indicate maximal FbFP intensities reached at the end of each culture. Black dots indicate the 36 individual cultivations. The upper x-axis reflects the corresponding optical density of the cultures at the time of induction. It is calculated from the mean scattered light values of the cultures that have not been induced until the respective induction time and a calibration curve that was previously prepared (see Additional file 1: Figure S5). Cultivation conditions for *E. coli* Tuner(DE3)/pRhotHi-2-EcFbFP: 800 µL Wilms-MOPS mineral medium per well in a 48-Flowerplate, sealed with a sandwich membrane (m2p-labs), 37 °C, shaking frequency 1400 rpm and shaking diameter 3 mm

The induction profile of *E. coli* Tuner(DE3) EcFbFP at 37 °C in a 48-well Flowerplate is illustrated in Fig. 2. During the whole cultivation process, scattered light and FbFP-fluorescence was measured for 36 individual cultivations (indicated by black dots in Fig. 2). The maximal achieved recombinant protein content in terms of fluorescence intensity at the end of each cultivation is shown in dependency of the inducer concentration and time of induction. Since many manuals recommend using 1 mM IPTG by default [13], the utilized IPTG gradient ranges from 0 to 1 mM. As described in the methods section, this IPTG-gradient was automatically added by the RoboLector into the 48-well Flowerplate. Thereby, the shaking process of the Flowerplate inside the BioLector device stopped shortly at distinct induction times ranging from 1 to 10 h after the start of the main culture.

A characteristic optimum consisting of a horizontal and vertical part forming a reversed “L” is indicated by red color coding in the graph. When induction takes place within the small time slot of 8–9 h of cultivation, high fluorescence intensities are reached independent of IPTG concentration. When induction occurs within the first 9 h of cultivation, a specific low inducer concentration of 0.05 mM IPTG has to be used.

The biomass concentration has to reach a distinct threshold to withstand the metabolic burden induced by higher IPTG concentrations. The higher the biomass concentration, the lower the IPTG to cell ratio. Involving the optical density OD_600_ (Fig. 2, upper x-axis), the threshold lies in this case at a value around 3.5. However, a very late induction is also counterproductive since most of the resources are then already converted to biomass instead of recombinant proteins. For practical and economic reasons, it is worthwhile to utilize low inducer concentrations (0.05 mM IPTG at 37 °C). This saves expensive chemicals and no mandatory online-monitoring is necessary since induction time has a low impact. However, it has to be mentioned that the same low IPTG concentration in a culture of strains holding the *lacY* gene could result in lower product formation due to the irregular distribution of IPTG within the entire population. Since the temperature is reduced in the next section, the large experimental space up to 1 mM IPTG was also checked at 30 °C (see Additional file 1: Figure S1). Just as for 37 °C, no improvement in product formation is observed for IPTG concentrations above 0.4 mM. To optimize the experimental search space, the maximum applied inducer concentration was therefore reduced to 0.4 mM IPTG. Consequently, higher information density is achieved within the inducer concentration ranging from 0 to 0.4 mM.

### Comparison of selected induction conditions at 28 and 37 °C

**Fig. 3:**
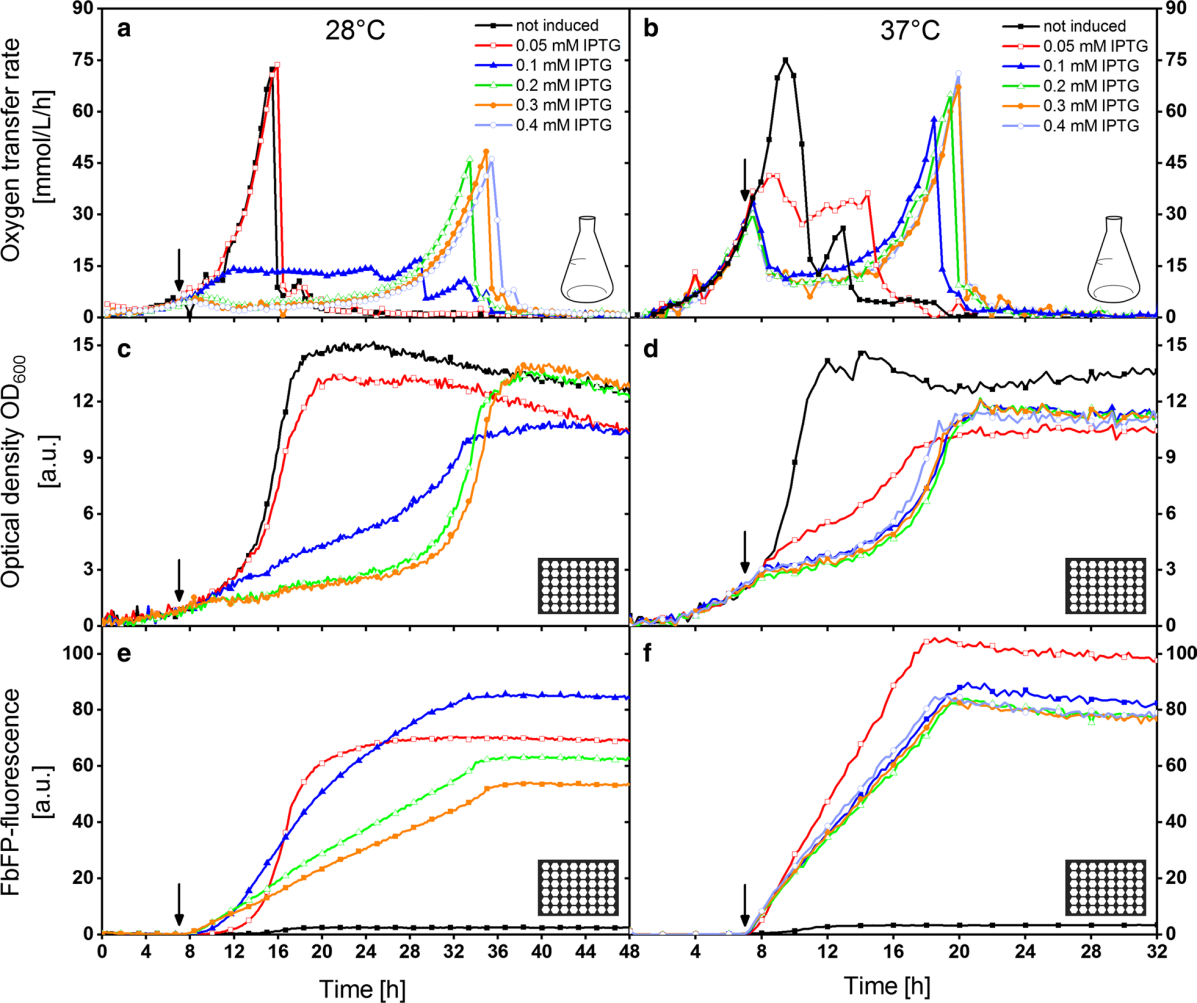
Comparison of selected induction conditions at 28 and 37 °C measured in a RAMOS and a BioLector device. Manual induction (shake flask) and automated induction (MTP) after 7 h with 0–0.4 mM IPTG (indicated by black arrows). Cultivation temperature was 28 °C for the graphs in the left column and 37 °C for the graphs in the right column. RAMOS and BioLector cultivations were conducted for each temperature in parallel using the same *mastermix* (medium plus microorganisms). **a**, **b** Oxygen-transfer rate of RAMOS cultivations in 250 mL shake flasks. **c**, **d** Scattered light signal from BioLector cultivations. **e**, **f** FbFP-fluorescence from BioLector cultivations. Cultivation conditions for *E. coli* Tuner(DE3)/pRhotHi-2-EcFbFP in RAMOS device: 8 mL Wilms-MOPS mineral medium in each 250 mL flask, shaking frequency 350 rpm, shaking diameter 50 mm, in BioLector device: 800 µL Wilms-MOPS mineral medium per well in a 48-Flowerplate, sealed with a sandwich membrane (m2p-labs), shaking frequency 1400 rpm and shaking diameter 3 mm

A comparison of OTR, biomass and product formation at 28 and 37 °C is given in Fig. 3. The standard cultivation temperature of *E. coli* is 37 °C. In contrast, 28 °C is far from the optimal growth temperature. However, it is often used to obtain soluble recombinant protein production and to avoid inclusion body formation [18, 20]. The results are obtained from two parallel RAMOS and BioLector cultivations, respectively. The same data are also available for 30 and 34 °C in Additional file 1: Figure S3. The cultivation at 37 °C was conducted under the same cultivation conditions as for Fig. 2, besides a smaller IPTG concentration range. Induction was manually performed in shake flask cultivations and automatically in microtiter plate cultivations after 7 h (indicated by arrows). This time was chosen in advance. It was intended to detect the most diverse oxygen transfer rates and product formations for different inducer concentrations at one induction time. Because of one failed automated induction in the microtiter plate, the curves for 0.4 mM IPTG at 28 °C (Fig. 3c, e) are missing.

The non-induced culture reaches the maximum OTR within 16 h at 28 °C (Fig. 3a), whereas it takes only 9 h at 37 °C (Fig. 3b). This clearly indicates, as expected, a higher growth rate at a higher temperature. For the induced cultures, the induction took place after 7 h for both temperatures. Therefore, the culture at 28 °C was induced at lower cell densities than the one at 37 °C. A direct comparison of the effect of induction at the same biomass concentration is provided in Additional file 1: Figure S4.

Surprisingly, induction with 0.05 mM IPTG at 28 °C hardly affects the respiration of *E. coli*, whereas at 37 °C, the OTR stagnates at a more or less constant level between 30 and 40 mmol/L/h until all of the carbon source is depleted and the OTR drops down. This phenomenon is also reflected in the optical density (Fig. 3c, d). The induced growth curve (0.05 mM IPTG) at 28 °C has only low deviations compared to the non-induced culture. The deviation becomes apparent at the end of the cultivation when an optical density of only 13, instead of 15, is reached. Thereby, some of the glucose is consumed to form the product instead of biomass. However, at 37 °C, the biomass growth is strongly disturbed after induction with 0.05 mM IPTG and only a very low final optical density of around 10 is reached. The cells obviously redirected key metabolites into the production of FbFP. This becomes clearly apparent in Fig. 3e, f. The induced culture (0.05 mM IPTG) at 37 °C results in higher fluorescent intensities than that at 28 °C. Moreover, the product formation seems to be linear at 37 °C and S-shaped at 28 °C. Generally, the production of FbFP stops for all cultivations as soon as the stationary phase is reached. Although the biomass concentration during induction is higher at 37 °C (lower inducer per biomass ratio), the metabolic burden is higher than for 28 °C.

Doubling the inducer concentration to 0.1 mM IPTG increases the metabolic burden significantly for both temperatures. The OTR curve at 28 °C also reveals a plateau, but at a lower level of around 13 mmol/L/h. The optical density progression looks similar to the cultivation with 0.05 mM IPTG at 37 °C, resulting in both having the highest production among the investigated induction conditions in Fig. 3. The induction with 0.1–0.4 mM IPTG at 37 °C lead to the following analogous trends. Directly after induction, the respiration is strongly reduced and recovers after a few hours, ending with a final relatively high OTR peak. This is consistent with the biomass growth, which is slower compared to 0.05 mM IPTG. The final product concentration attains only a value of 80 a.u. Increasing the IPTG concentration to 0.2 mM or higher at 28 °C, results in similar trends. The OTR stays at a very low level below 4 mmol/L/h after induction, the optical density barely increases for a long time and only low final product concentrations are achieved. However, the biomass growth recovers after 28 h and high final ODs are reached; thus, marginal energy is spent for recombinant protein production. Thus, the metabolic burden was too high at the time of induction.

The results are in good agreement with previously published observations. Kunze et al. noticed a correlation of OTR and product formation. Phases of low OTR were identified as production phases whereas phases of high OTR were related to low productivity and undisturbed cell growth [47]. There is one extension, which has to be made; phases of low OTR are only related to high productivity if a specific threshold is exceeded. A respiration rate of about 10 mmol/L/h should still be present.

### Comparison of induction profiles at four temperatures

**Fig. 4:**
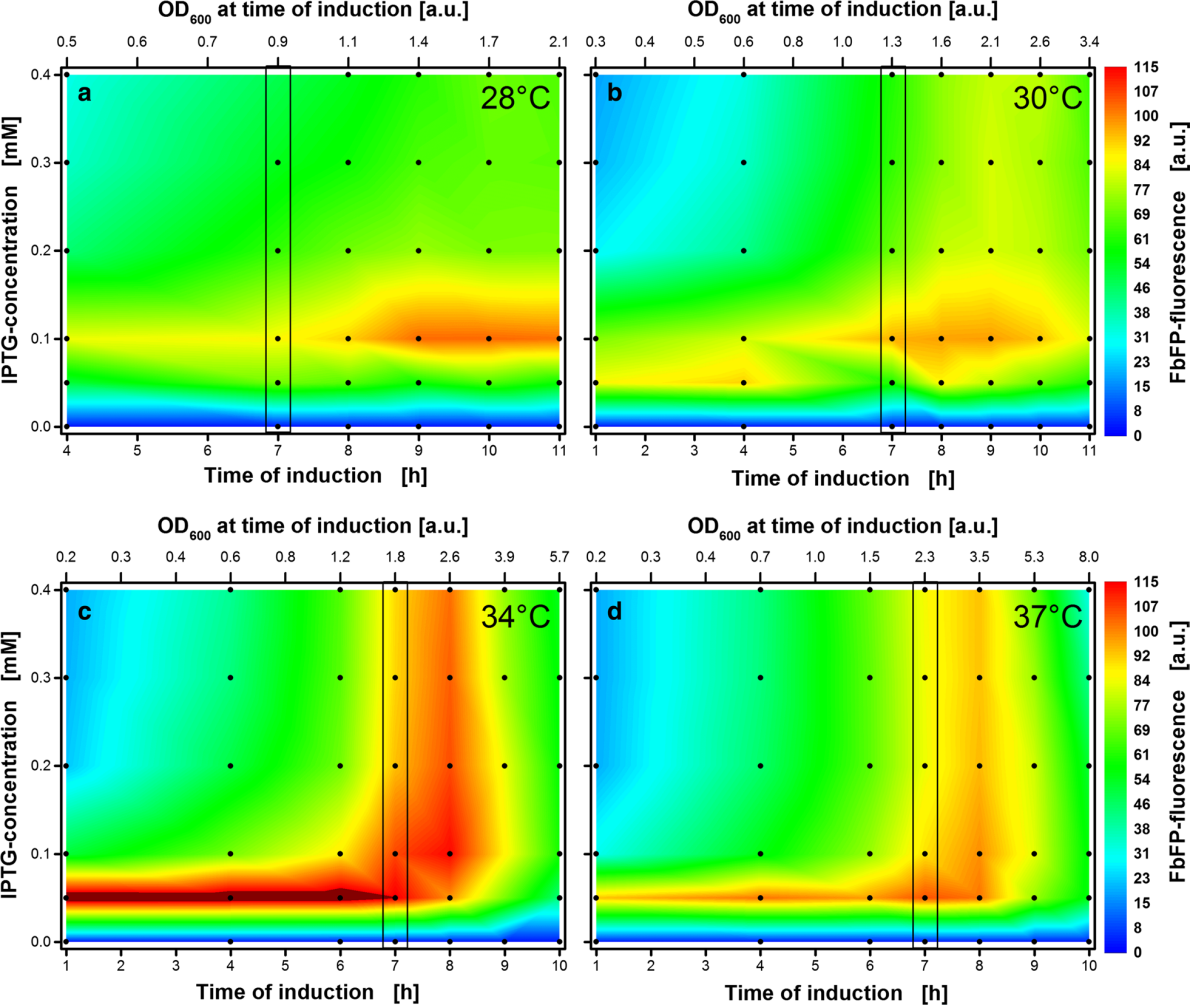
Induction profiles with automated addition of 0–0.4 mM IPTG at different temperatures. Colors from blue to red indicate maximal FbFP intensities reached at the end of each culture. Black dots indicate the 35–42 individual cultivations for each temperature. The upper x-axis reflect the corresponding optical density of the cultures at the time of induction. It is calculated from the mean scattered light values of cultures that have not been induced until the respective induction time and a calibration curve that was previously prepared (see Additional file 1: Figure S5). Black boxes indicate conditions of parallel RAMOS cultivations. **a** 28 °C, induction after 4–11 h. **b** 30 °C, induction after 1–11 h. **c** 34 °C, induction after 1–10 h. **d** 37 °C, induction after 1–10 h. Cultivation conditions for *E. coli* Tuner(DE3)/pRhotHi-2-EcFbFP: 800 µL Wilms-MOPS mineral medium per well in a 48-Flowerplate sealed, with a sandwich membrane (m2p-labs), shaking frequency 1400 rpm and shaking diameter 3 mm

So far, a strong influence of temperature on the metabolism of *E. coli* Tuner(DE3) pRhotHi-2-EcFbFP was presented in the previous section. The results were shown for selected induction conditions as time progressed. Figure 4 shows the overall formed product at four temperatures (28, 30, 34 and 37 °C) over a broad range of induction conditions. The corresponding induction profiles are illustrated in Fig. 4a–d. Vertical black boxes symbolize the induction conditions from parallel shake flask cultivations. Thereby, Fig. 4a, d refer to Fig. 3 and Fig. 4b, c refer to Additional file 1: Figure S3.

The induction profile at 37 °C (Fig. 4d) is a replication of Fig. 2 at lower IPTG concentrations. The area for optimal induction concurs quite well for both experiments. The ideal inducer concentration of 0.05 mM IPTG is identical as well as the best time-point for induction at 8 h or accordingly, an optical density of 3.5.

With a reduction in temperature, the optimal induction zone is shifted and altered (Fig. 4a–d). Firstly, the absolute measured product concentration varies with temperature. The horizontal optimum at 34 °C (0.05 mM IPTG, 1–6 h) is even outside of the applied fluorescence scale. Since the FbFP fluorescence is not temperature sensitive within the investigated temperature range and no inclusion body formation at 37 °C could be detected via SDS-PAGE (data not shown), the highest product concentration is achieved at 34 °C. These findings are in contradiction with the common approach to reduce temperature for increased productivity [20], but are consistent with some other studies [33, 34].

Secondly, the vertical optimum strongly changes. While the vertical optimal zone is present at 37 and 34 °C, it fades out at 30 °C and disappears at 28 °C. Thus, a defined low inducer concentration is all the more important at lower temperatures. There was the assumption of a later vertical optimum (induction after 11 h and later) for 28 and 30 °C but it was rebutted by the trend of lower optimal optical densities at lower temperatures. While the best optical density for induction is 3.5 (x-axis at the top of the graph) at 37 °C, it decreases to 2.6 at 34 °C. It is less clear at 30 °C, but there is a light green-yellow area above the optimum, in particular at 9 h. This less pronounced vertical area corresponds to an optical density of 2.1, which is again lower compared to the higher temperatures. Therefore, a further optimum after 11 h at 28 °C is rather unlikely.

Thirdly, the optimal inducer concentration decreases with higher temperatures. This effect was already identified above (see Fig. 3). Thus, the trend is valid for a broad range of induction conditions. While the optimal inducer concentration is 0.1 mM IPTG at 28 °C, it decreases at 34 and 37 °C to 0.05 mM IPTG. A transition area is visible at 30 °C, where 0.05 mM IPTG is preferable for early induction and 0.1 mM IPTG is better for later induction. The optimal inducer concentration seems to lie in between the two concentrations. Thus, this area is investigated in more detail in the next section.

Interestingly, the induction profiles are in good accordance with experiments conducted in 96-well plates (see Additional file 1: Figure S2). The same trends can be identified for 30 and 37 °C. Also, the latter discussed effect of alternating optimal IPTG concentration is visible in the induction profile at 30 °C (Additional file 1: Figure S2C). However, the absolute FbFP fluorescence intensities cannot be compared since completely different well geometries and filling volumes were used in 96-well plates.

### Induction profile with low inducer concentrations

**Fig. 5:**
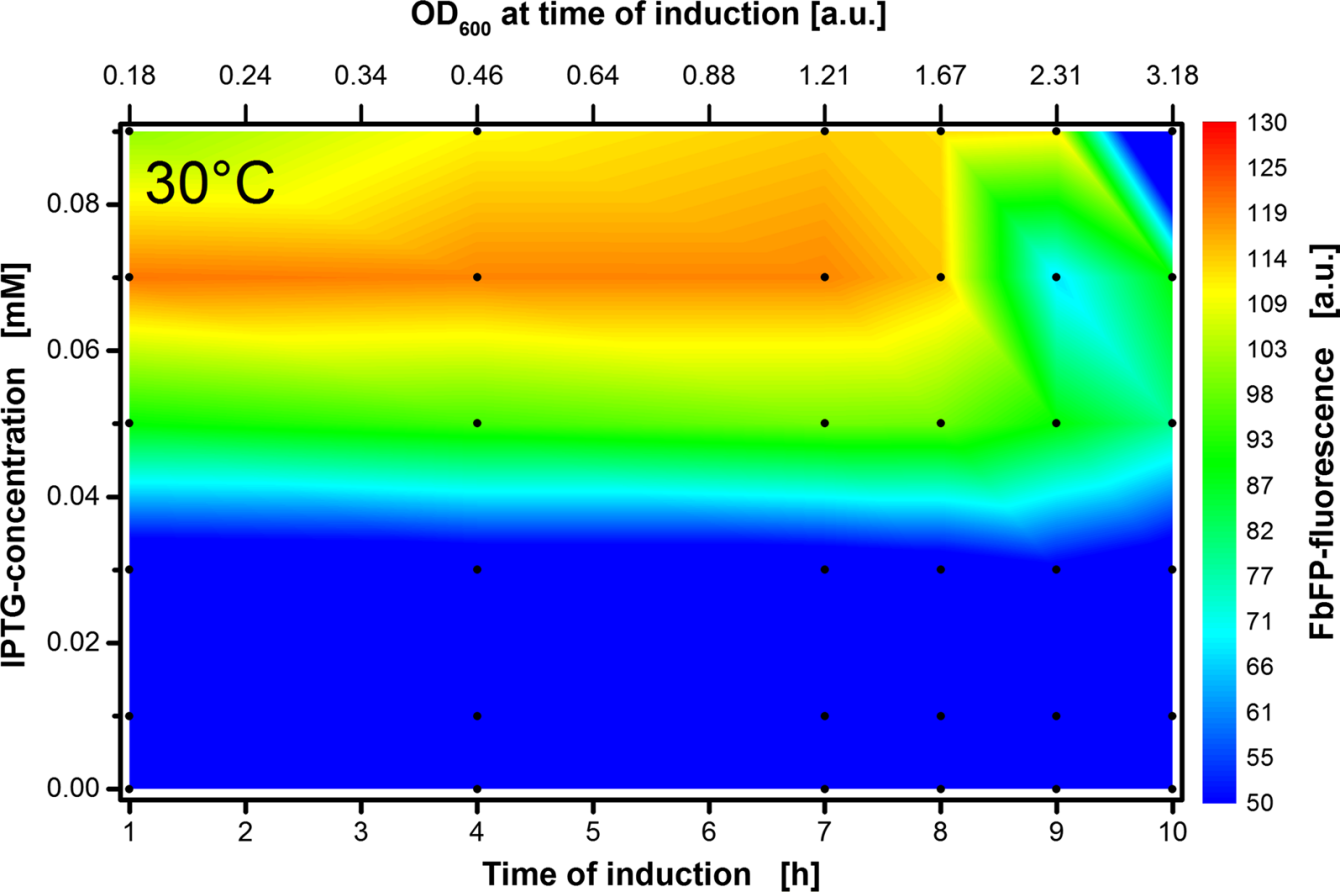
Induction profile with automated addition of 0–0.09 mM IPTG solution after 1–10 h of cultivation time. Colors from blue to red indicate maximal FbFP intensities reached at the end of each culture. Black dots indicate the 36 individual cultivations. The upper x-axis reflects the corresponding optical density of the cultures at the time of induction. It is calculated from the mean scattered light values of cultures that have not been induced until the respective induction time and a calibration curve that was previously prepared (see Additional file 1: Figure S5). Cultivation conditions for *E. coli* Tuner(DE3)/pRhotHi-2-EcFbFP: 800 µL Wilms-MOPS mineral medium per well in a 48-Flowerplate, sealed with a sandwich membrane (m2p-labs), 30 °C, shaking frequency 1400 rpm and shaking diameter 3 mm

In the previous section, a switch in the optimal inducer concentration from 0.05 to 0.1 mM was detected at 30 °C. It was assumed that the real optimal inducer concentration is to be found between those concentrations. In order to examine the optimal induction conditions at 30 °C more closely, a detailed analysis of smaller inducer concentrations ranging from 0 to 0.09 mM IPTG was conducted. The results are presented in Fig. 5. To highlight the best induction conditions, the scale indicating the FbFP fluorescence is modified in this plot. The highest FbFP fluorescence intensity of 120 a.u. is reached by induction with 0.07 mM IPTG within the first 8 h. The best inducer concentration of 0.05 and 0.1 mM found in the upper section (see Fig. 4b) results only in a maximal fluorescence intensity of 100 a.u. Thus, already very small changes in IPTG concentration can alter the outcome, which is why the overall product formation could be further enhanced by a fine-tuning of the inducer concentration. The highest FbFP fluorescence of 125 a.u. identified in the previous section at 34 °C (0.05 mM IPTG—Fig. 4c) can now be explained differently. Since the product concentration is in the same range as for the improved protocol at 30 °C, the assumption is obvious, that the ideal inducer concentration was coincidently met at 34 °C. Probably, equally high product formation can be achieved at all temperatures between 28 and 37 °C when the exact individual inducer concentration between 0 and 0.1 mM IPTG is applied.

## Conclusion

Within this study, the ideal induction conditions for the expression of recombinant proteins as a function of temperature for the *lacY* mutant strain *E. coli* Tuner(DE3) expressing the fluorescent protein EcFbFP was comprehensively investigated. In comparison to other strains, the lack of lactose permease in this strain enables the uniform distribution of inducer concentration over the entire population. Automated induction profiles at temperatures of 28, 30, 34, and 37 °C were prepared in non-oxygen limited cultures in 48-well Flowerplates using a robotic platform. The obtained results clearly illustrate major changes in induction optima at different temperatures. In general, the following trends could be determined:

- Optimal inducer concentrations for the investigated strain were far below the conventional recommended inducer concentration of 1 mM IPTG. The best results were obtained with an IPTG concentration between 0.05 and 0.1 mM for all investigated temperatures.
- The higher the temperature, the lower the optimal IPTG concentration.
- The lower the temperature, the smaller the scope for inducer concentration. At higher temperatures, the area for optimal induction formed a reversed “L”, meaning a horizontal optimum at different induction times and a vertical optimum at different inducer concentrations. At lower temperatures, only the horizontal optimum is left. While IPTG concentrations from 0.05 to 1 mM IPTG at distinct induction times result in high product formation at 37 °C, it is restricted to 0.1 mM at 28 °C.

For selected induction conditions, parallel shake flask cultivations were conducted to evaluate the oxygen transfer rates. Comparable cultivation conditions in microtiter plate and shake flask were guaranteed by keeping the maximum oxygen transfer capacity (OTR_max_) constant. The highest product formation was obtained, when the metabolic burden led to a reduced and relatively constant low respiration activity. Furthermore, this was related to a linear growth behavior [48]. When induction took place at the same biomass concentration, lower inducer concentrations resulted in similar metabolic burden, at higher temperatures than the higher inducer concentrations at lower temperatures. The validity of the above reported findings for other strains and promoter systems still has to be investigated. However, in preliminary experiments, the *E. coli* BL21(DE3) strain expressing a cellulase under the control of different promoters resulted also in high product concentrations when small inducer concentrations (0.05–0.1 mM) were applied.

These results confirm the need for induction profiling. Without precise investigation of induction conditions, it is almost impossible to find optimal cultivation conditions to express high amounts of recombinant proteins. Certainly, another *E. coli* strain or another target protein can lead to completely different induction profiles, but the effect of temperature should always be considered. When comparing expression efficiencies at different temperatures, the respective optimal induction conditions always have to be used. Otherwise, the comparison is misleading.

## Additional file

**Additional file 1: Figure S1.** Induction profile with automated addition of 0–1 mM IPTG solution after 1–10 h of cultivation time at 30 °C. **Figure S2.** Comparison of induction profiles at 30 and 37 °C in 48-well plates and 96-well plates. **Figure S3.** Comparison of selected induction conditions at 30 and 34 °C measured in a RAMOS and a BioLector device. **Figure S4.** Comparison of selected induction conditions at 30 and 34 °C measured in a RAMOS and a BioLector device. **Figure S5.** Calibration curves for conversion of scattered light measured by the RoboLector device to standard optical density in 48-well and 96-well plates.

## Abbreviations

a.u.: arbitrary unit
CTR: carbon dioxide transfer rate
DOT: dissolved oxygen tension
*E. coli*: *Escherichia coli*
FbFP: (FMN)-binding fluorescent protein
FMN: flavin mononucleotide
GFP: green fluorescent protein
IPTG: isopropyl β-d-thiogalactopyranoside
OD: optical density
OD_600_: optical density at 600 nm
OTR: oxygen transfer rate
OTR_max_: maximum oxygen transfer capacity
RAMOS: respiration activity monitoring system
RQ: respiratory quotient
TB: terrific broth
