# Supplementary material for: Optimizing recombinant protein expression via automated induction profiling in microtiter plates at different temperatures
Source: Microb Cell Fact. 2017 Nov 28;16:220. doi: 10.1186/s12934-017-0832-4 (PMC5706349; doi:10.1186/s12934-017-0832-4)
Supplement: Supplementary file 1 — Additional file 1: Figure S1. Induction profile with automated addition of 0–1 mM IPTG solution after 1–10 h of cultivation time at 30 °C. Figure S2. Comparison of induction profiles at 30 and 37 °C in 48-well plates and 96-well plates. Figure S3. Comparison of selected induction conditions at 30 and 34 °C measured in a RAMOS and a BioLector device. Figure S4. Comparison of selected induction conditions at 30 and 34 °C measured in a RAMOS and a BioLector device. Figure S5. Calibration curves for conversion of scattered light measured by the RoboLector device to standard optical density in 48-well and 96-well plates. [file 12934_2017_832_MOESM1_ESM.docx]

# Additional file 1





**Figure S1: Induction profile with automated addition of 0 - 1 mM IPTG solution after 1 - 10 h of cultivation time at 30°C.** Colors from blue to red indicate maximal reached FbFP intensities at the end of each culture. Black dots indicate the 36 individual cultivations. . The upper x-axis reflects the corresponding optical density of the cultures at the time of induction. It is calculated from the mean scattered light values of the cultures that have not been induced until the respective induction time and a calibration curve that was previously prepared (see **Additional file 1: Figure S5***)*. Cultivation conditions for *E. coli* Tuner(DE3)/pRhotHi-2-EcFbFP: 800 µL Wilms-MOPS mineral medium per well in a 48-Flower plate, sealed with a sandwich membrane (m2p-labs), 30°C, shaking frequency 1400 rpm, shaking diameter 3 mm.





**Figure S2: Comparison of induction profiles at 30°C and 37°C in 48-well plates and 96-well plates.** Colors from blue to red indicate maximal reached FbFP intensities at the end of each culture. Black dots indicate the 42-80 individual cultivations for each induction profile. The upper x-axes reflect the corresponding optical density of the cultures at the time of induction. It is calculated from the mean scattered light values of cultures that have not been induced until the respective induction time and a calibration curve that was previously prepared *(see Appendix)*. **A)** 30°C, 48-well plate, induction after 1 - 11 h, **B)** 37°C, 48-well plate, induction after 1 - 10 h, **C)** 30°C, 96-well plate, induction after 1 - 11 h, **D)** 37°C, 96-well plate, induction after 1 - 11 h. Cultivation conditions for *E. coli* Tuner(DE3)/pRhotHi-2-EcFbFP in 48-well plates: 800 µL Wilms-MOPS mineral medium per well in a 48-Flower Plate, sealed with a sandwich membrane (m2p-labs), shaking frequency 1400 rpm, shaking diameter 3 mm. For standard 96-well plates: 200 µL Wilms-MOPS mineral medium per well, sealed with AeraSeal membrane, shaking frequency 1000 rpm, shaking diameter 3 mm.





**Figure S3: Comparison of selected induction conditions at 30°C and 34°C measured in a RAMOS and a BioLector device.** Manual induction (shake flask) and automated induction (MTP) after 7 h with 0-0.4 mM IPTG (indicated by black arrow). Cultivation temperature was 30°C for the graphs in the left column and 34°C for the graphs in the right column. RAMOS and BioLector cultivations were conducted for each temperature in parallel using the same ‘mastermix’ (medium plus microorganisms). **A), B)** Oxygen-transfer rate of RAMOS cultivations in 250 mL shake flasks, **C), D)** Scattered light signal from BioLector cultivations, **E), F)** FbFP-fluorescence. Cultivation conditions for *E. coli* Tuner(DE3)/pRhotHi-2-EcFbFP in RAMOS device: 8 mL Wilms-MOPS mineral medium in each flask, shaking frequency 350 rpm, shaking diameter 50 mm, in BioLector device: 800 µL Wilms-MOPS mineral medium per well in a 48-Flower Plate, sealed with a sandwich membrane (m2p-labs), shaking frequency 1400 rpm, shaking diameter 3 mm.





**Figure S4: Comparison of selected induction conditions at 28°C and 37°C at same optical density measured in BioLector device.** Automated induction after 11 h (A, C) and 7 h (B, D) with 0 - 0.4 mM IPTG (indicated by black arrow). Dotted line visualizes induction at same biomass concentration. Cultivation temperature was 28°C for the graphs in the left column and 37°C for the graphs in the right column. **A), B)** Scattered light signal and **C), D)** FbFP-fluorescence. Cultivation conditions for *E. coli* Tuner(DE3)/pRhotHi-2-EcFbFP in BioLector device: 800 µL Wilms-MOPS mineral medium per well in a 48-Flower Plate sealed with a sandwich membrane (m2p-labs), shaking frequency 1400 rpm, shaking diameter 3 mm.

**

**

**Figure S5: Calibration curves for conversion of scattered light measured by the RoboLector device to standard optical density in 48-well and 96-well plates.** Calibration was performed with cells from stationary phase. Optical densities from 0.1-12 were prepared. Standard deviations are only available for 96-well plates and were derived from three independent measurements. Equations from linear fits can be found in the figure. Cultivation conditions: *E. coli* Tuner(DE3)/pRhotHi-2-EcFbFP dilutions were measured in BioLector device: 800 µL filling volume in a 48-Flower plate sealed with a sandwich membrane (m2p-labs), 28-37°C, shaking frequency 1400 rpm, shaking diameter 3 mm. 200 µL filling volume in a 96-well plate, sealed with AeraSeal membrane, 28/37°C shaking frequency, 1000 rpm, shaking diameter 3 mm.
